# Correction: Aleemardani et al. Graphene-Based Materials Prove to Be a Promising Candidate for Nerve Regeneration Following Peripheral Nerve Injury. *Biomedicines* 2022, *10*, 73

**DOI:** 10.3390/biomedicines13071711

**Published:** 2025-07-14

**Authors:** Mina Aleemardani, Pariya Zare, Amelia Seifalian, Zohreh Bagher, Alexander M. Seifalian

**Affiliations:** 1 Biomaterials and Tissue Engineering Group, Department of Materials Science and Engineering, Kroto Research Institute, The University of Sheffield, Sheffield S3 7HQ, UK; maleemardani1@sheffield.ac.uk; 2 Department of Chemical Engineering, University of Tehran, Tehran 1417935840, Iran; pariyazare@ut.ac.ir; 3 Department of Surgery and Cancer, Imperial College London, London W12 0NN, UK; a.seifalian@ic.ac.uk; 4 ENT and Head and Neck Research Centre, Hazrat Rasoul Akram Hospital, The Five Senses Health Institute, Iran University of Medical Sciences, Tehran 16844, Iran; 5 Nanotechnology and Regenerative Medicine Commercialization Centre (NanoRegMed Ltd.), London BioScience Innovation Centre, London NW1 0NH, UK

**Update to Conflicts of Interest**

There was an error in the original publication [1]. Alexander M. Seifalian and Amelia Seifalian did not declare the disclose a conflict of interest regarding their work on material developed by NanoRegMed Ltd.

A correction has been made to Conflicts of Interest Statement as below:

Alexander M. Seifalian is the co-founder and scientific director of NanoRegMed Ltd., as indicated in the affiliation. He and his research team have developed and patented a graphene-based nanocomposite material; trade registered as BioHastalex^®^. This has been highlighted in the manuscript as their current work in London. Amelia Seifalian is associated with NanoRegMed but does not hold a formal position or receive financial compensation from the company.

The authors state that the scientific conclusions are unaffected. This correction was approved by the Academic Editor. The original publication has also been updated.
